# 1-Acetyl-5-ferrocenyl-3-phenyl-2-pyrazoline

**DOI:** 10.1107/S1600536808043298

**Published:** 2009-01-08

**Authors:** Nevzat Karadayı, Günseli Turgut Cin, Seda Demirel, Abban Çakıcı, Orhan Büyükgüngör

**Affiliations:** a Samsun Vocational School, Ondokuz Mayıs University, TR-55139, Samsun, Turkey; b Department of Chemistry, Faculty of Arts and Sciences, Akdeniz University, TR-07058, Antalya, Turkey; c Department of Physics, Faculty of Arts and Sciences, Ondokuz Mayıs University, TR-55139, Samsun, Turkey

## Abstract

In the title compound, [Fe(C_5_H_5_)(C_16_H_15_N_2_O)], the pyrazoline ring and the phenyl ring are nearly coplanar, making a dihedral angle of 6.54 (2)°, while the substituted cyclo­penta­dienyl ring is twisted out of the pyrazoline ring plane by 81.32 (1)°. The mol­ecules in the crystal structure are held together by weak C—H⋯O inter­molecular hydrogen bonds and two C—H⋯π inter­actions.

## Related literature

For background to the applications of pyrazolines, see: Amr *et al.* (2006 ▶); Biot *et al.* (2004 ▶); Fang *et al.* (2003 ▶); Fouda *et al.* (2007 ▶); Guirado *et al.* (2004 ▶); Jaouen *et al.* (2004 ▶); Johnson *et al.* (2007 ▶); Küçükgüzel *et al.* (2000 ▶); Karthikeyan *et al.* (2007 ▶); Özdemir *et al.* (2007 ▶). For bond-length data, see: Jian *et al.* (2008 ▶). For related structures, see: Turgut Cin *et al*. (2008 ▶); Kudar *et al.* (2005 ▶); Köysal *et al.* (2005 ▶).
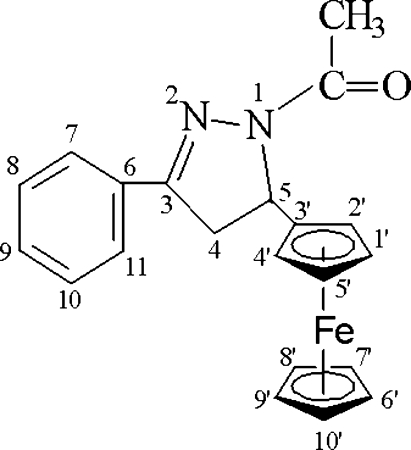


## Experimental

### 

#### Crystal data

[Fe(C_5_H_5_)(C_16_H_15_N_2_O)]
*M* *_r_* = 372.24
Monoclinic, 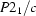

*a* = 6.0762 (4) Å
*b* = 43.155 (2) Å
*c* = 7.3512 (4) Å
β = 116.218 (4)°
*V* = 1729.33 (17) Å^3^
*Z* = 4
Mo *K*α radiation
μ = 0.88 mm^−1^
*T* = 296 (2) K
0.49 × 0.33 × 0.05 mm

#### Data collection

STOE IPDS 2 diffractometer
Absorption correction: integration (*X-RED32*; Stoe & Cie, 2002 ▶) *T*_min_ = 0.662, *T*_max_ = 0.962
15964 measured reflections
3256 independent reflections
2765 reflections with *I* > 2σ(*I*)
*R*_int_ = 0.045

#### Refinement

*R*[*F* ^2^ > 2σ(*F* ^2^)] = 0.035
*wR*(*F* ^2^) = 0.093
*S* = 1.04
3256 reflections
227 parameters
1 restraint
H-atom parameters constrained
Δρ_max_ = 0.23 e Å^−3^
Δρ_min_ = −0.46 e Å^−3^

### 

Data collection: *X-AREA* (Stoe & Cie, 2002 ▶); cell refinement: *X-AREA*; data reduction: *X-RED* (Stoe & Cie, 2002 ▶); program(s) used to solve structure: *SHELXS97* (Sheldrick, 2008 ▶); program(s) used to refine structure: *SHELXL97* (Sheldrick, 2008 ▶); molecular graphics: *ORTEPIII* (Farrugia, 1997 ▶); software used to prepare material for publication: *WinGX* (Farrugia, 1999 ▶) and *PLATON* (Spek, 2003 ▶).

## Supplementary Material

Crystal structure: contains datablocks I. DOI: 10.1107/S1600536808043298/at2695sup1.cif

Structure factors: contains datablocks I. DOI: 10.1107/S1600536808043298/at2695Isup2.hkl

Additional supplementary materials:  crystallographic information; 3D view; checkCIF report

## Figures and Tables

**Table 1:** Hydrogen-bond geometry (Å, °)

*D*—H⋯*A*	*D*—H	H⋯*A*	*D*⋯*A*	*D*—H⋯*A*
C5—H5⋯O1^i^	0.93	2.70	3.599 (3)	163 (1)
C16—H16⋯O1^ii^	0.93	2.29	3.201 (3)	167 (1)
C17—H17⋯*Cg*1^iii^	0.93	2.71	3.64 (4)	174 (1)
C11—H11*A*⋯*Cg*2^iv^	0.96	2.60	3.51 (5)	158 (1)

Symmetry codes: (i) 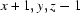; (ii) 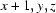; (iii) 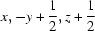; (iv) 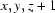. *Cg*1 and *Cg*2 are the centroids of the C17—C21 and C1—C6 rings, respectively.

**Fig. 1.**
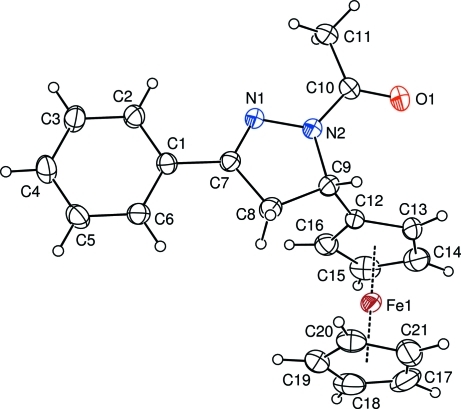
A view of (I), with the atom-numbering scheme. Displacement ellipsoids are drawn at the 30% probability.

**Fig. 2.**
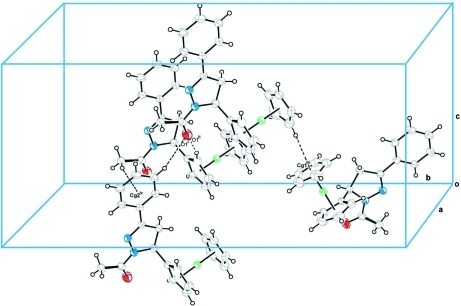
The crystal structure of (I), showing both C—H···O intermolecular hydrogen bonds and C—H···π interactions as dashed lines. H atoms not involved in hydrogen bonds have been omitted for clarity [symmetry codes: (iii) x, 1/2 - y, 1/2 + z; (iv) x, y, 1 + z].

## supplementary crystallographic information

### Comment

Pyrazolines are well known nitrogen-containing five-membered heterocyclic compounds. Condensation of nitrogen-containing binucleophilic agents with α, β unsaturated ketones is one of the most suitable synthetic pathways for 2-pyrazolines (Kudar *et al.*, 2005), which possess widespread pharmaceutical properties such as antimicrobial (Küçükgüzel *et al.*, 2000), anticonvulsant (Karthikeyan *et al.*, 2007), antidepressant (Özdemir *et al.*, 2007), antiandrogenic (Amr *et al.*, 2006), antifungal and anti-inflammatory (Guirado *et al.*, 2004) activities. Furthermore, *N*-acetylated 2-pyrazolines are inhibitors of kinesin spindle protein (KSP); potentially useful for the treatment cancer (Johnson *et al.*, 2007). Metallocenes are also known to exhibit a wide range of biological activity. Among them ferrocenyl compounds display interesting antibacterial (Fouda *et al.*, 2007), antitumor (Jaouen *et al.*, 2004), antimalarial and antifungal (Biot *et al.*, 2004) activities. Therefore, incorporation of a ferrocene fragment into a heterocyclic ring may enhance their biological activities or generate new medicinal properties (Fang *et al.*, 2003). As a part of an ongoing investigation of the chemistry of ferrocenyl pyrazolines, the title compound (I) was synthesized and its crystal structure was determined.

The molecular structure of the title compound is shown in Fig. 1. The dihedral angle of 6.54 (2)° between pyrazoline ring and the phenyl ring indicates that they are conjugated with each other; this is accord with the C1—C7 bond [1.474 (3) Å], which has double-bond character (Jian *et al.*, 2008). Furthermore, N1—C7 bond length [1.286 (3) Å] increased as a result of this conjugation. This observation is in good agreement with those reported for 1-acetyl-3-ferrocenyl-5-(2-nitrophenyl)-2-pyrazoline (Turgut Cin *et al.*, 2008), 5-ferrocenyl-3-(*p*-methoxyphenyl)-1-(2-pyridyl) -2-pyrazoline (Kudar *et al.*, 2005) and 3-(4-fluorophenyl)-*N*-methyl-5- (4-methylphenyl)-2-pyrazoline-1-thiocarboxamide (Köysal *et al.*, 2005).

The Fe—*Cg*_s_ and Fe—*Cg*_as_ distances are 1.6454 (13)Å and 1.6510 (15) Å, respectively, and the *Cg*_s_—Fe—*Cg*_as_ angle is 178.90 (8)°, where *Cg*_s_ and *Cg*_as_ are the centroids of the substituted and unsubstituted Cp rings. The small dihedral angle of 3.2963 (2)° between the unsubstituted and substituted Cp rings exposes that the two Cp rings are parallel to each other. The average C12—*Cg*_s_—*Cg*_as_—C20 torsion angle of 4.789 (2)° brings that the two Cp rings of the ferrocenyl group is nearly in an eclipsed conformation.

The pyrazoline ring and substituted Cp ring make a dihedral angle of 81.32 (1)°. The dihedral angle between the phenyl ring and substituted Cp ring is 75.82 (1)°, whereas the phenyl ring plane deviates from the unsubstituted Cp ring with an angle of 76.60 (1)°. The molecules in the crystal held together by two weak intermolecular C5—H5···O1 and C16—H16···O1 hydrogen bonds and two C—H···π interactions (Table 1, Fig. 2).

### Experimental

A mixture of 3-ferrocenyl-1-phenyl-propen-2-one (0.32 mmol, 0.1 g), 80% hydrazine monohydrate (7.04 mmol, 0.45 g) and glacial acetic acid (10 ml) was refluxed under nitrogen atmosphere for 4 h. TLC indicated the formation of the reaction product. It was poured into ice-water to give orange solid. The participate was separated by filtration and washed with water. The solid product was dried at room temperature. Single crystals of the title compound suitable for X-ray measurements were obtained by recrystallization from methanol at room temperature (Yield 82%; m.p. 456–457 K). IR (KBr, cm^-1^): 1647 (C=O), 1596 (C=N), 1580 (C=C), 1102 (C—N), 507 (Cp—Fe—Cp). ^1^H-NMR (CDCl_3_, p.p.m..): 2.33 (s, 1H, CH_3_), 3.50 (dd, 1H, pyr.), 3.68 (dd, 1H, pyr.), 4.02 (s, 1H, Fc), 4.12 (s, 1H, Fc), 4.16 (s, 5H, Fc), 4.18 (s, 1H, Fc), 4.51 (s, 1H, Fc), 5.51 (dd, 1H, pyr.), 7.47–7.81 (m, 5H, Arom.). ^13^C-NMR (CDCl_3_, p.p.m.): 22.04 (CH_3_), 39.54 (pyr. CH_2_), 55.37 (pyr. CH), 65.57 (C_Fc_), 68.22 (C_Fc_), 68.37 (C_Fc_), 68.58 (C_Fc_), 70.34 (C_Fc_), 87.38 (C_Fcipso_), 126.53 (C_phenyl_), 128.81 (C_phenyl_), 130.27 (C_phenyl_), 131.59 (C_phenyl_), 153.88 (pyr. C=N), 168.87 (C=O).

### Refinement

All C—H atoms were refined using the riding model approximation, with C—H = 0.93–0.98Å [*U*_iso_(H) = 1.2 or 1.5*U*_eq_(C)].

### Crystal data

**Table:**

[Fe(C_5_H_5_)(CH_15_N_2_O)]	*F*(000) = 776
*M**_r_* = 372.24	*D*_x_ = 1.430 Mg m^−^^3^
Monoclinic, *P*2_1_/*c*	Mo *K*α radiation, λ = 0.71073 Å
Hall symbol: -P 2ybc	Cell parameters from 15964 reflections
*a* = 6.0762 (4) Å	θ = 1.9–26.2°
*b* = 43.155 (2) Å	µ = 0.88 mm^−^^1^
*c* = 7.3512 (4) Å	*T* = 296 K
β = 116.218 (4)°	Prism, brown
*V* = 1729.33 (17) Å^3^	0.49 × 0.33 × 0.05 mm
*Z* = 4	

### Data collection

**Table:**

STOE IPDS 2 diffractometer	3256 independent reflections
Radiation source: fine-focus sealed tube	2765 reflections with *I* > 2σ(*I*)
plane graphite	*R*_int_ = 0.045
ω scans	θ_max_ = 25.7°, θ_min_ = 2.8°
Absorption correction: integration (*X-RED32*; Stoe & Cie, 2002)	*h* = −7→7
*T*_min_ = 0.662, *T*_max_ = 0.962	*k* = −52→51
15964 measured reflections	*l* = −8→8

### Refinement

**Table:**

Refinement on *F*^2^	Primary atom site location: structure-invariant direct methods
Least-squares matrix: full	Secondary atom site location: difference Fourier map
*R*[*F*^2^ > 2σ(*F*^2^)] = 0.035	Hydrogen site location: inferred from neighbouring sites
*wR*(*F*^2^) = 0.093	H-atom parameters constrained
*S* = 1.04	*w* = 1/[σ^2^(*F*_o_^2^) + (0.0493*P*)^2^ + 0.5221*P*] where *P* = (*F*_o_^2^ + 2*F*_c_^2^)/3
3256 reflections	(Δ/σ)_max_ = 0.002
227 parameters	Δρ_max_ = 0.23 e Å^−^^3^
1 restraint	Δρ_min_ = −0.46 e Å^−^^3^

### Special details

**Table:**

Geometry. All e.s.d.'s (except the e.s.d. in the dihedral angle between two l.s. planes) are estimated using the full covariance matrix. The cell e.s.d.'s are taken into account individually in the estimation of e.s.d.'s in distances, angles and torsion angles; correlations between e.s.d.'s in cell parameters are only used when they are defined by crystal symmetry. An approximate (isotropic) treatment of cell e.s.d.'s is used for estimating e.s.d.'s involving l.s. planes.
Refinement. Refinement of *F*^2^ against ALL reflections. The weighted *R*-factor *wR* and goodness of fit *S* are based on *F*^2^, conventional *R*-factors *R* are based on *F*, with *F* set to zero for negative *F*^2^. The threshold expression of *F*^2^ > σ(*F*^2^) is used only for calculating *R*-factors(gt) *etc*. and is not relevant to the choice of reflections for refinement. *R*-factors based on *F*^2^ are statistically about twice as large as those based on *F*, and *R*- factors based on ALL data will be even larger.

### Fractional atomic coordinates and isotropic or equivalent isotropic displacement parameters (Å^2^)

**Table:**

	*x*	*y*	*z*	*U*_iso_*/*U*_eq_	
C1	0.4032 (4)	0.43314 (5)	0.3252 (3)	0.0411 (4)	
C2	0.4385 (4)	0.46493 (5)	0.3407 (3)	0.0495 (5)	
H2	0.3732	0.4766	0.4115	0.059*	
C3	0.5694 (5)	0.47951 (6)	0.2524 (3)	0.0579 (6)	
H3	0.5932	0.5008	0.2645	0.069*	
C4	0.6652 (5)	0.46210 (6)	0.1453 (4)	0.0588 (6)	
H4	0.7585	0.4717	0.0897	0.071*	
C5	0.6226 (4)	0.43077 (6)	0.1213 (3)	0.0559 (6)	
H5	0.6812	0.4193	0.0446	0.067*	
C6	0.4926 (4)	0.41618 (5)	0.2109 (3)	0.0482 (5)	
H6	0.4648	0.3949	0.1947	0.058*	
C7	0.2756 (4)	0.41761 (5)	0.4301 (3)	0.0406 (4)	
C8	0.2110 (5)	0.38395 (5)	0.4107 (4)	0.0542 (5)	
H8A	0.3558	0.3711	0.4491	0.065*	
H8B	0.0940	0.3788	0.2732	0.065*	
C9	0.0970 (4)	0.37977 (5)	0.5595 (3)	0.0458 (5)	
H9	−0.0725	0.3725	0.4857	0.055*	
C10	−0.0242 (4)	0.42188 (5)	0.7322 (3)	0.0482 (5)	
C11	−0.0126 (4)	0.45555 (6)	0.7829 (4)	0.0547 (6)	
H11A	0.1361	0.4597	0.9028	0.066*	
H11B	−0.1511	0.4610	0.8065	0.066*	
H11C	−0.0153	0.4676	0.6722	0.066*	
C12	0.2353 (4)	0.35877 (5)	0.7359 (3)	0.0426 (4)	
C13	0.1310 (5)	0.33836 (6)	0.8274 (4)	0.0541 (5)	
H13	−0.0358	0.3356	0.7874	0.065*	
C14	0.3218 (6)	0.32297 (7)	0.9888 (4)	0.0700 (7)	
H14	0.3034	0.3080	1.0722	0.084*	
C15	0.5448 (5)	0.33420 (7)	1.0017 (4)	0.0711 (7)	
H15	0.7001	0.3282	1.0969	0.085*	
C16	0.4944 (4)	0.35620 (6)	0.8458 (4)	0.0554 (5)	
H16	0.6099	0.3670	0.8198	0.067*	
C17	0.3595 (9)	0.26768 (7)	0.6984 (6)	0.0991 (13)	
H17	0.3663	0.2531	0.7939	0.119*	
C18	0.5592 (7)	0.28122 (7)	0.6853 (5)	0.0803 (9)	
H18	0.7234	0.2774	0.7713	0.096*	
C19	0.4730 (5)	0.30115 (6)	0.5240 (4)	0.0611 (6)	
H19	0.5706	0.3129	0.4824	0.073*	
C20	0.2195 (5)	0.30119 (7)	0.4321 (4)	0.0634 (6)	
H20	0.1183	0.3129	0.3201	0.076*	
C21	0.1421 (7)	0.28010 (8)	0.5394 (6)	0.0942 (12)	
H21	−0.0188	0.2753	0.5118	0.113*	
N1	0.2119 (3)	0.43277 (4)	0.5499 (2)	0.0415 (4)	
N2	0.0981 (3)	0.41220 (4)	0.6266 (3)	0.0460 (4)	
O1	−0.1350 (3)	0.40299 (4)	0.7846 (3)	0.0664 (5)	
Fe1	0.34526 (6)	0.314282 (7)	0.72673 (4)	0.04627 (12)	

### Atomic displacement parameters (Å^2^)

**Table:**

	*U*^11^	*U*^22^	*U*^33^	*U*^12^	*U*^13^	*U*^23^
C1	0.0476 (10)	0.0436 (11)	0.0351 (9)	0.0025 (8)	0.0209 (8)	0.0023 (8)
C2	0.0659 (13)	0.0453 (13)	0.0471 (11)	−0.0044 (10)	0.0339 (11)	−0.0033 (9)
C3	0.0803 (15)	0.0497 (14)	0.0555 (13)	−0.0140 (12)	0.0407 (12)	−0.0041 (10)
C4	0.0671 (14)	0.0682 (17)	0.0538 (13)	−0.0073 (12)	0.0382 (11)	0.0038 (11)
C5	0.0633 (14)	0.0658 (16)	0.0511 (12)	0.0108 (11)	0.0365 (11)	0.0039 (11)
C6	0.0597 (12)	0.0431 (12)	0.0472 (11)	0.0074 (9)	0.0286 (10)	0.0035 (9)
C7	0.0484 (10)	0.0365 (11)	0.0395 (10)	0.0026 (8)	0.0220 (9)	0.0023 (8)
C8	0.0817 (15)	0.0400 (12)	0.0555 (12)	−0.0002 (11)	0.0436 (12)	−0.0006 (10)
C9	0.0549 (12)	0.0370 (11)	0.0526 (11)	−0.0039 (9)	0.0301 (10)	−0.0027 (9)
C10	0.0479 (11)	0.0500 (13)	0.0550 (12)	0.0037 (9)	0.0305 (10)	0.0021 (10)
C11	0.0625 (13)	0.0508 (14)	0.0627 (13)	0.0073 (11)	0.0385 (12)	−0.0016 (11)
C12	0.0514 (11)	0.0360 (11)	0.0504 (11)	−0.0037 (8)	0.0316 (9)	−0.0050 (8)
C13	0.0717 (14)	0.0464 (13)	0.0640 (14)	−0.0035 (11)	0.0480 (12)	0.0000 (11)
C14	0.112 (2)	0.0588 (16)	0.0556 (14)	0.0114 (15)	0.0519 (15)	0.0085 (12)
C15	0.0776 (17)	0.0735 (19)	0.0495 (13)	0.0159 (15)	0.0165 (12)	−0.0036 (13)
C16	0.0543 (12)	0.0516 (14)	0.0614 (13)	−0.0045 (10)	0.0264 (11)	−0.0113 (11)
C17	0.195 (4)	0.0351 (15)	0.106 (3)	0.004 (2)	0.102 (3)	0.0044 (15)
C18	0.102 (2)	0.0650 (19)	0.0792 (18)	0.0324 (17)	0.0446 (17)	0.0048 (15)
C19	0.0748 (16)	0.0577 (15)	0.0647 (14)	0.0012 (12)	0.0434 (13)	−0.0073 (12)
C20	0.0726 (16)	0.0580 (16)	0.0581 (14)	0.0058 (12)	0.0274 (12)	−0.0140 (12)
C21	0.104 (2)	0.075 (2)	0.141 (3)	−0.0429 (19)	0.088 (3)	−0.059 (2)
N1	0.0475 (9)	0.0362 (9)	0.0476 (9)	0.0001 (7)	0.0273 (8)	0.0034 (7)
N2	0.0572 (10)	0.0357 (9)	0.0577 (10)	−0.0002 (8)	0.0369 (9)	0.0011 (8)
O1	0.0712 (11)	0.0596 (11)	0.0951 (13)	−0.0052 (8)	0.0611 (10)	0.0010 (9)
Fe1	0.0639 (2)	0.03565 (19)	0.04901 (19)	0.00135 (13)	0.03384 (16)	0.00207 (13)

### Geometric parameters (Å, °)

**Table:**

C1—C2	1.385 (3)	C12—Fe1	2.044 (2)
C1—C6	1.393 (3)	C13—C14	1.406 (4)
C1—C7	1.474 (3)	C13—Fe1	2.043 (2)
C2—C3	1.381 (3)	C13—H13	0.9300
C2—H2	0.9300	C14—C15	1.402 (4)
C3—C4	1.388 (3)	C14—Fe1	2.029 (2)
C3—H3	0.9300	C14—H14	0.9300
C4—C5	1.373 (4)	C15—C16	1.414 (4)
C4—H4	0.9300	C15—Fe1	2.032 (3)
C5—C6	1.384 (3)	C15—H15	0.9300
C5—H5	0.9300	C16—Fe1	2.039 (2)
C6—H6	0.9300	C16—H16	0.9300
C7—N1	1.286 (3)	C17—C18	1.388 (5)
C7—C8	1.495 (3)	C17—C21	1.426 (5)
C8—C9	1.542 (3)	C17—Fe1	2.028 (3)
C8—H8A	0.9700	C17—H17	0.9300
C8—H8B	0.9700	C18—C19	1.368 (4)
C9—N2	1.483 (3)	C18—Fe1	2.042 (3)
C9—C12	1.498 (3)	C18—H18	0.9300
C9—H9	0.9800	C19—C20	1.382 (4)
C10—O1	1.223 (3)	C19—Fe1	2.041 (2)
C10—N2	1.356 (3)	C19—H19	0.9300
C10—C11	1.494 (3)	C20—C21	1.414 (4)
C11—H11A	0.9600	C20—Fe1	2.033 (2)
C11—H11B	0.9600	C20—H20	0.9300
C11—H11C	0.9600	C21—Fe1	2.023 (3)
C12—C13	1.417 (3)	C21—H21	0.9300
C12—C16	1.421 (3)	N1—N2	1.389 (2)
			
C2—C1—C6	118.84 (19)	C21—C17—H17	126.1
C2—C1—C7	120.36 (18)	Fe1—C17—H17	125.7
C6—C1—C7	120.8 (2)	C19—C18—C17	108.3 (3)
C3—C2—C1	120.9 (2)	C19—C18—Fe1	70.41 (15)
C3—C2—H2	119.6	C17—C18—Fe1	69.52 (17)
C1—C2—H2	119.6	C19—C18—H18	125.9
C2—C3—C4	119.5 (2)	C17—C18—H18	125.9
C2—C3—H3	120.2	Fe1—C18—H18	125.8
C4—C3—H3	120.2	C18—C19—C20	110.0 (3)
C5—C4—C3	120.2 (2)	C18—C19—Fe1	70.45 (16)
C5—C4—H4	119.9	C20—C19—Fe1	69.86 (14)
C3—C4—H4	119.9	C18—C19—H19	125.0
C4—C5—C6	120.1 (2)	C20—C19—H19	125.0
C4—C5—H5	120.0	Fe1—C19—H19	126.3
C6—C5—H5	120.0	C19—C20—C21	107.4 (3)
C5—C6—C1	120.3 (2)	C19—C20—Fe1	70.49 (14)
C5—C6—H6	119.8	C21—C20—Fe1	69.23 (17)
C1—C6—H6	119.8	C19—C20—H20	126.3
N1—C7—C1	120.89 (19)	C21—C20—H20	126.3
N1—C7—C8	114.44 (17)	Fe1—C20—H20	125.6
C1—C7—C8	124.66 (17)	C20—C21—C17	106.5 (3)
C7—C8—C9	103.14 (17)	C20—C21—Fe1	69.96 (15)
C7—C8—H8A	111.1	C17—C21—Fe1	69.55 (18)
C9—C8—H8A	111.1	C20—C21—H21	126.8
C7—C8—H8B	111.1	C17—C21—H21	126.8
C9—C8—H8B	111.1	Fe1—C21—H21	125.3
H8A—C8—H8B	109.1	C7—N1—N2	107.90 (17)
N2—C9—C12	111.39 (17)	C10—N2—N1	122.19 (18)
N2—C9—C8	100.85 (16)	C10—N2—C9	123.93 (17)
C12—C9—C8	115.38 (18)	N1—N2—C9	113.44 (15)
N2—C9—H9	109.6	C21—Fe1—C17	41.22 (16)
C12—C9—H9	109.6	C21—Fe1—C14	120.23 (13)
C8—C9—H9	109.6	C17—Fe1—C14	107.75 (12)
O1—C10—N2	119.5 (2)	C21—Fe1—C15	154.41 (16)
O1—C10—C11	122.8 (2)	C17—Fe1—C15	119.21 (15)
N2—C10—C11	117.72 (19)	C14—Fe1—C15	40.41 (12)
C10—C11—H11A	109.5	C21—Fe1—C20	40.80 (13)
C10—C11—H11B	109.5	C17—Fe1—C20	68.15 (13)
H11A—C11—H11B	109.5	C14—Fe1—C20	155.83 (13)
C10—C11—H11C	109.5	C15—Fe1—C20	162.88 (12)
H11A—C11—H11C	109.5	C21—Fe1—C16	163.86 (15)
H11B—C11—H11C	109.5	C17—Fe1—C16	153.23 (16)
C13—C12—C16	107.3 (2)	C14—Fe1—C16	68.46 (11)
C13—C12—C9	126.1 (2)	C15—Fe1—C16	40.64 (11)
C16—C12—C9	126.57 (19)	C20—Fe1—C16	126.18 (11)
C13—C12—Fe1	69.67 (13)	C21—Fe1—C19	67.35 (11)
C16—C12—Fe1	69.46 (13)	C17—Fe1—C19	66.57 (12)
C9—C12—Fe1	127.31 (14)	C14—Fe1—C19	162.48 (12)
C14—C13—C12	108.6 (2)	C15—Fe1—C19	126.26 (12)
C14—C13—Fe1	69.26 (14)	C20—Fe1—C19	39.65 (11)
C12—C13—Fe1	69.76 (12)	C16—Fe1—C19	108.62 (10)
C14—C13—H13	125.7	C21—Fe1—C18	68.04 (15)
C12—C13—H13	125.7	C17—Fe1—C18	39.88 (15)
Fe1—C13—H13	126.9	C14—Fe1—C18	125.98 (12)
C15—C14—C13	107.8 (2)	C15—Fe1—C18	107.74 (13)
C15—C14—Fe1	69.91 (14)	C20—Fe1—C18	67.11 (12)
C13—C14—Fe1	70.32 (13)	C16—Fe1—C18	119.50 (13)
C15—C14—H14	126.1	C19—Fe1—C18	39.14 (12)
C13—C14—H14	126.1	C21—Fe1—C13	108.82 (11)
Fe1—C14—H14	125.2	C17—Fe1—C13	127.20 (13)
C14—C15—C16	108.7 (2)	C14—Fe1—C13	40.41 (11)
C14—C15—Fe1	69.68 (16)	C15—Fe1—C13	67.68 (11)
C16—C15—Fe1	69.97 (14)	C20—Fe1—C13	121.85 (11)
C14—C15—H15	125.6	C16—Fe1—C13	68.10 (10)
C16—C15—H15	125.6	C19—Fe1—C13	156.12 (11)
Fe1—C15—H15	126.3	C18—Fe1—C13	163.57 (12)
C15—C16—C12	107.6 (2)	C21—Fe1—C12	126.67 (14)
C15—C16—Fe1	69.40 (15)	C17—Fe1—C12	164.70 (15)
C12—C16—Fe1	69.80 (12)	C14—Fe1—C12	68.53 (10)
C15—C16—H16	126.2	C15—Fe1—C12	68.28 (10)
C12—C16—H16	126.2	C20—Fe1—C12	108.77 (10)
Fe1—C16—H16	126.2	C16—Fe1—C12	40.74 (9)
C18—C17—C21	107.8 (3)	C19—Fe1—C12	121.34 (10)
C18—C17—Fe1	70.60 (18)	C18—Fe1—C12	154.21 (12)
C21—C17—Fe1	69.23 (17)	C13—Fe1—C12	40.57 (8)
C18—C17—H17	126.1		
			
C6—C1—C2—C3	−2.9 (3)	C16—C15—Fe1—C17	156.81 (18)
C7—C1—C2—C3	176.4 (2)	C16—C15—Fe1—C14	−120.0 (2)
C1—C2—C3—C4	0.5 (4)	C14—C15—Fe1—C20	165.3 (3)
C2—C3—C4—C5	2.3 (4)	C16—C15—Fe1—C20	45.3 (4)
C3—C4—C5—C6	−2.7 (4)	C14—C15—Fe1—C16	120.0 (2)
C4—C5—C6—C1	0.3 (3)	C14—C15—Fe1—C19	−164.32 (16)
C2—C1—C6—C5	2.5 (3)	C16—C15—Fe1—C19	75.72 (19)
C7—C1—C6—C5	−176.8 (2)	C14—C15—Fe1—C18	−125.14 (19)
C2—C1—C7—N1	−5.5 (3)	C16—C15—Fe1—C18	114.90 (17)
C6—C1—C7—N1	173.87 (19)	C14—C15—Fe1—C13	38.05 (16)
C2—C1—C7—C8	175.1 (2)	C16—C15—Fe1—C13	−81.90 (16)
C6—C1—C7—C8	−5.6 (3)	C14—C15—Fe1—C12	81.97 (17)
N1—C7—C8—C9	−2.9 (3)	C16—C15—Fe1—C12	−37.99 (14)
C1—C7—C8—C9	176.64 (19)	C19—C20—Fe1—C21	−118.3 (3)
C7—C8—C9—N2	4.2 (2)	C19—C20—Fe1—C17	−79.2 (2)
C7—C8—C9—C12	−115.9 (2)	C21—C20—Fe1—C17	39.1 (2)
N2—C9—C12—C13	101.6 (2)	C19—C20—Fe1—C14	−164.0 (2)
C8—C9—C12—C13	−144.2 (2)	C21—C20—Fe1—C14	−45.6 (4)
N2—C9—C12—C16	−76.6 (3)	C19—C20—Fe1—C15	39.8 (4)
C8—C9—C12—C16	37.6 (3)	C21—C20—Fe1—C15	158.1 (4)
N2—C9—C12—Fe1	−167.51 (13)	C19—C20—Fe1—C16	74.78 (19)
C8—C9—C12—Fe1	−53.3 (2)	C21—C20—Fe1—C16	−166.9 (2)
C16—C12—C13—C14	−1.0 (3)	C21—C20—Fe1—C19	118.3 (3)
C9—C12—C13—C14	−179.5 (2)	C19—C20—Fe1—C18	−35.92 (18)
Fe1—C12—C13—C14	58.49 (17)	C21—C20—Fe1—C18	82.4 (2)
C16—C12—C13—Fe1	−59.53 (15)	C19—C20—Fe1—C13	159.63 (16)
C9—C12—C13—Fe1	122.0 (2)	C21—C20—Fe1—C13	−82.0 (2)
C12—C13—C14—C15	1.4 (3)	C19—C20—Fe1—C12	116.80 (16)
Fe1—C13—C14—C15	60.16 (18)	C21—C20—Fe1—C12	−124.9 (2)
C12—C13—C14—Fe1	−58.80 (16)	C15—C16—Fe1—C21	162.8 (4)
C13—C14—C15—C16	−1.2 (3)	C12—C16—Fe1—C21	44.0 (4)
Fe1—C14—C15—C16	59.26 (18)	C15—C16—Fe1—C17	−49.7 (3)
C13—C14—C15—Fe1	−60.42 (18)	C12—C16—Fe1—C17	−168.6 (2)
C14—C15—C16—C12	0.5 (3)	C15—C16—Fe1—C14	37.14 (16)
Fe1—C15—C16—C12	59.60 (15)	C12—C16—Fe1—C14	−81.68 (15)
C14—C15—C16—Fe1	−59.08 (19)	C12—C16—Fe1—C15	−118.8 (2)
C13—C12—C16—C15	0.3 (3)	C15—C16—Fe1—C20	−164.97 (16)
C9—C12—C16—C15	178.8 (2)	C12—C16—Fe1—C20	76.21 (16)
Fe1—C12—C16—C15	−59.34 (17)	C15—C16—Fe1—C19	−124.45 (17)
C13—C12—C16—Fe1	59.67 (15)	C12—C16—Fe1—C19	116.73 (14)
C9—C12—C16—Fe1	−121.9 (2)	C15—C16—Fe1—C18	−83.01 (19)
C21—C17—C18—C19	−0.5 (3)	C12—C16—Fe1—C18	158.16 (14)
Fe1—C17—C18—C19	−60.0 (2)	C15—C16—Fe1—C13	80.79 (17)
C21—C17—C18—Fe1	59.5 (2)	C12—C16—Fe1—C13	−38.03 (12)
C17—C18—C19—C20	0.6 (3)	C15—C16—Fe1—C12	118.8 (2)
Fe1—C18—C19—C20	−58.80 (19)	C18—C19—Fe1—C21	−82.6 (2)
C17—C18—C19—Fe1	59.4 (2)	C20—C19—Fe1—C21	38.6 (2)
C18—C19—C20—C21	−0.5 (3)	C18—C19—Fe1—C17	−37.6 (2)
Fe1—C19—C20—C21	−59.63 (18)	C20—C19—Fe1—C17	83.5 (2)
C18—C19—C20—Fe1	59.1 (2)	C18—C19—Fe1—C14	36.8 (5)
C19—C20—C21—C17	0.1 (3)	C20—C19—Fe1—C14	157.9 (3)
Fe1—C20—C21—C17	−60.3 (2)	C18—C19—Fe1—C15	72.4 (2)
C19—C20—C21—Fe1	60.43 (18)	C20—C19—Fe1—C15	−166.49 (17)
C18—C17—C21—C20	0.2 (3)	C18—C19—Fe1—C20	−121.1 (3)
Fe1—C17—C21—C20	60.56 (19)	C18—C19—Fe1—C16	114.1 (2)
C18—C17—C21—Fe1	−60.3 (2)	C20—C19—Fe1—C16	−124.73 (17)
C1—C7—N1—N2	−179.60 (17)	C20—C19—Fe1—C18	121.1 (3)
C8—C7—N1—N2	−0.1 (2)	C18—C19—Fe1—C13	−168.0 (3)
O1—C10—N2—N1	175.1 (2)	C20—C19—Fe1—C13	−46.9 (3)
C11—C10—N2—N1	−6.1 (3)	C18—C19—Fe1—C12	157.19 (19)
O1—C10—N2—C9	3.2 (3)	C20—C19—Fe1—C12	−81.69 (19)
C11—C10—N2—C9	−177.9 (2)	C19—C18—Fe1—C21	80.7 (2)
C7—N1—N2—C10	−169.37 (19)	C17—C18—Fe1—C21	−38.5 (2)
C7—N1—N2—C9	3.3 (2)	C19—C18—Fe1—C17	119.2 (3)
C12—C9—N2—C10	−69.3 (3)	C19—C18—Fe1—C14	−167.12 (18)
C8—C9—N2—C10	167.7 (2)	C17—C18—Fe1—C14	73.7 (3)
C12—C9—N2—N1	118.16 (18)	C19—C18—Fe1—C15	−126.2 (2)
C8—C9—N2—N1	−4.8 (2)	C17—C18—Fe1—C15	114.6 (2)
C20—C21—Fe1—C17	−117.2 (2)	C19—C18—Fe1—C20	36.36 (18)
C20—C21—Fe1—C14	160.19 (16)	C17—C18—Fe1—C20	−82.8 (2)
C17—C21—Fe1—C14	−82.6 (2)	C19—C18—Fe1—C16	−83.5 (2)
C20—C21—Fe1—C15	−165.3 (2)	C17—C18—Fe1—C16	157.3 (2)
C17—C21—Fe1—C15	−48.0 (3)	C17—C18—Fe1—C19	−119.2 (3)
C17—C21—Fe1—C20	117.2 (2)	C19—C18—Fe1—C13	162.8 (4)
C20—C21—Fe1—C16	41.2 (5)	C17—C18—Fe1—C13	43.6 (5)
C17—C21—Fe1—C16	158.4 (4)	C19—C18—Fe1—C12	−49.6 (4)
C20—C21—Fe1—C19	−37.48 (16)	C17—C18—Fe1—C12	−168.8 (2)
C17—C21—Fe1—C19	79.8 (2)	C14—C13—Fe1—C21	114.8 (2)
C20—C21—Fe1—C18	−79.93 (18)	C12—C13—Fe1—C21	−124.90 (19)
C17—C21—Fe1—C18	37.32 (18)	C14—C13—Fe1—C17	72.5 (2)
C20—C21—Fe1—C13	117.28 (17)	C12—C13—Fe1—C17	−167.26 (19)
C17—C21—Fe1—C13	−125.47 (19)	C12—C13—Fe1—C14	120.3 (2)
C20—C21—Fe1—C12	75.59 (19)	C14—C13—Fe1—C15	−38.04 (18)
C17—C21—Fe1—C12	−167.16 (17)	C12—C13—Fe1—C15	82.21 (16)
C18—C17—Fe1—C21	118.7 (3)	C14—C13—Fe1—C20	157.98 (18)
C18—C17—Fe1—C14	−125.4 (2)	C12—C13—Fe1—C20	−81.76 (17)
C21—C17—Fe1—C14	115.90 (19)	C14—C13—Fe1—C16	−82.06 (18)
C18—C17—Fe1—C15	−82.8 (2)	C12—C13—Fe1—C16	38.19 (14)
C21—C17—Fe1—C15	158.42 (18)	C14—C13—Fe1—C19	−168.7 (3)
C18—C17—Fe1—C20	80.0 (2)	C12—C13—Fe1—C19	−48.5 (3)
C21—C17—Fe1—C20	−38.75 (17)	C14—C13—Fe1—C18	38.8 (5)
C18—C17—Fe1—C16	−48.1 (3)	C12—C13—Fe1—C18	159.1 (4)
C21—C17—Fe1—C16	−166.9 (2)	C14—C13—Fe1—C12	−120.3 (2)
C18—C17—Fe1—C19	36.91 (19)	C13—C12—Fe1—C21	75.45 (19)
C21—C17—Fe1—C19	−81.83 (19)	C16—C12—Fe1—C21	−166.07 (16)
C21—C17—Fe1—C18	−118.7 (3)	C9—C12—Fe1—C21	−45.1 (2)
C18—C17—Fe1—C13	−165.84 (18)	C13—C12—Fe1—C17	41.7 (5)
C21—C17—Fe1—C13	75.4 (2)	C16—C12—Fe1—C17	160.2 (4)
C18—C17—Fe1—C12	161.2 (3)	C9—C12—Fe1—C17	−78.8 (5)
C21—C17—Fe1—C12	42.5 (5)	C13—C12—Fe1—C14	−36.99 (16)
C15—C14—Fe1—C21	157.8 (2)	C16—C12—Fe1—C14	81.49 (16)
C13—C14—Fe1—C21	−83.8 (2)	C9—C12—Fe1—C14	−157.5 (2)
C15—C14—Fe1—C17	114.5 (2)	C13—C12—Fe1—C15	−80.60 (17)
C13—C14—Fe1—C17	−127.1 (2)	C16—C12—Fe1—C15	37.89 (15)
C13—C14—Fe1—C15	118.4 (2)	C9—C12—Fe1—C15	158.9 (2)
C15—C14—Fe1—C20	−169.5 (2)	C13—C12—Fe1—C20	117.39 (15)
C13—C14—Fe1—C20	−51.0 (3)	C16—C12—Fe1—C20	−124.12 (14)
C15—C14—Fe1—C16	−37.34 (16)	C9—C12—Fe1—C20	−3.2 (2)
C13—C14—Fe1—C16	81.07 (16)	C13—C12—Fe1—C16	−118.49 (19)
C15—C14—Fe1—C19	46.4 (4)	C9—C12—Fe1—C16	121.0 (2)
C13—C14—Fe1—C19	164.8 (3)	C13—C12—Fe1—C19	159.21 (15)
C15—C14—Fe1—C18	74.2 (2)	C16—C12—Fe1—C19	−82.30 (16)
C13—C14—Fe1—C18	−167.35 (17)	C9—C12—Fe1—C19	38.7 (2)
C15—C14—Fe1—C13	−118.4 (2)	C13—C12—Fe1—C18	−166.6 (3)
C15—C14—Fe1—C12	−81.29 (17)	C16—C12—Fe1—C18	−48.1 (3)
C13—C14—Fe1—C12	37.13 (14)	C9—C12—Fe1—C18	72.9 (3)
C14—C15—Fe1—C21	−49.1 (4)	C16—C12—Fe1—C13	118.49 (19)
C16—C15—Fe1—C21	−169.0 (3)	C9—C12—Fe1—C13	−120.6 (2)
C14—C15—Fe1—C17	−83.2 (2)		

### Hydrogen-bond geometry (Å, °)

**Table:**

*D*—H···*A*	*D*—H	H···*A*	*D*···*A*	*D*—H···*A*
C5—H5···O1^i^	0.93	2.70	3.599 (3)	163 (1)
C16—H16···O1^ii^	0.93	2.29	3.201 (3)	167 (1)
C17—H17···Cg1^iii^	0.93	2.71	3.64 (4)	174 (1)
C11—H11A···Cg2^iv^	0.96	2.60	3.51 (5)	158 (1)

Symmetry codes: (i) *x*+1, *y*, *z*−1; (ii) *x*+1, *y*, *z*; (iii) *x*, −*y*+1/2, *z*+1/2; (iv) *x*, *y*, *z*+1.
